# Supplementary material for: Transcriptomic Insights into Mechanisms of Early Seed Maturation in the Garden Pea (Pisum sativum L.)
Source: Cells. 2020 Mar 23;9(3):779. doi: 10.3390/cells9030779 (PMC7140803; doi:10.3390/cells9030779)

# Sprint−2(20) vs Zhongv

## Global Summary

%DE = 0.79

# genes with fdr < 0.2 = 1582 ( 743 + / 839 −)

# genes with fdr < 0.1 = 1390 ( 638 + / 752 −)

# genes with fdr < 0.05 = 1144 ( 507 + / 637 −)

# genes with fdr < 0.01 = 756 ( 310 + / 446 −)

# genes in genesets = 0

<FC> = 0

<t−score> = −2.35

<p−value> = 0.01

<fdr> = 0.21

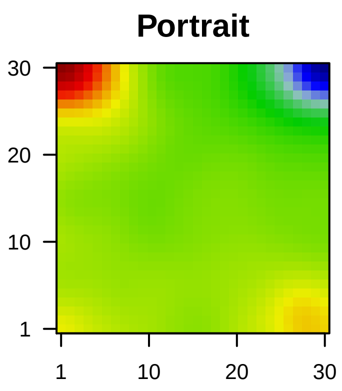


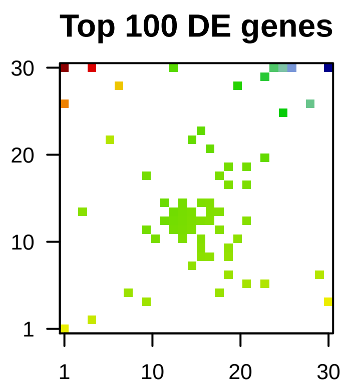


## Global Genelist

| Rank | ID | log(FC) | p−value | fdr | Metagene | Description |
|---|---|---|---|---|---|---|
| 1 | TRINITY_DN4 | −0.1 | 2e−11 | 0.0000000[illegible] | [illegible] x 5 | |
| 2 | TRINITY_DN8 | −3.19 | 4e−10 | 0.0000000[illegible] | [illegible] x 30 | |
| 3 | TRINITY_DN9 | −0.01 | 4e−10 | 0.0000003[illegible] | [illegible] x 14 | |
| 4 | TRINITY_DN2 | −0.01 | 1e−09 | 0.0000005[illegible] | [illegible] x 13 | |
| 5 | TRINITY_DN1 | −0.01 | 3e−09 | 0.0000005[illegible] | [illegible] x 13 | |
| 6 | TRINITY_DN7 | −0.03 | 6e−09 | 0.0000002[illegible] | [illegible] x 20 | |
| 7 | TRINITY_DN2 | −0.03 | 7e−09 | 0.0000002[illegible] | [illegible] x 11 | |
| 8 | TRINITY_DN2 | −0.01 | 8e−09 | 0.0000005[illegible] | [illegible] x 14 | |
| 9 | TRINITY_DN1 | −0.01 | 9e−09 | 0.0000005[illegible] | [illegible] x 13 | |
| 10 | TRINITY_DN1 | −0.01 | 9e−09 | 0.0000005[illegible] | [illegible] x 13 | |
| 11 | TRINITY_DN9 | −0.01 | 1e−08 | 0.0000005[illegible] | [illegible] x 12 | |
| 12 | TRINITY_DN1 | −0.01 | 1e−08 | 0.0000005[illegible] | [illegible] x 12 | |
| 13 | TRINITY_DN5 | −0.01 | 1e−08 | 0.0000005[illegible] | [illegible] x 9 | |
| 14 | TRINITY_DN8 | −0.07 | 1e−08 | 0.0000007[illegible] | [illegible] x 22 | |
| 15 | TRINITY_DN2 | −0.02 | 2e−08 | 0.0000007[illegible] | [illegible] x 18 | |
| 16 | TRINITY_DN7 | 0 | 2e−08 | 0.0000007[illegible] | [illegible] x 14 | |
| 17 | TRINITY_DN9 | −0.01 | 2e−08 | 0.0000014[illegible] | [illegible] x 12 | |
| 18 | TRINITY_DN1 | −0.01 | 3e−08 | 0.0000014[illegible] | [illegible] x 5 | |
| 19 | TRINITY_DN2 | −0.02 | 3e−08 | 0.0000013[illegible] | [illegible] x 14 | |
| 20 | TRINITY_DN4 | −0.01 | 3e−08 | 0.0000014[illegible] | [illegible] x 11 | |

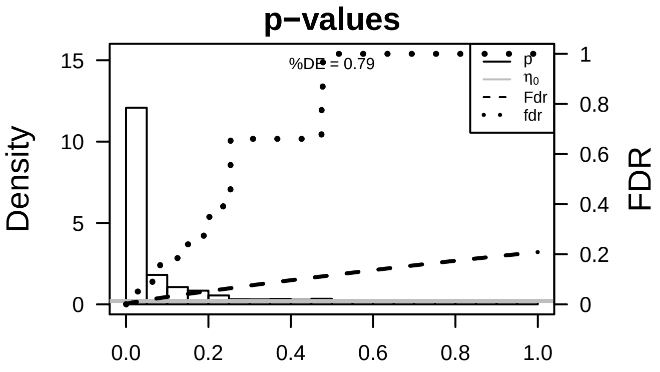

Supplement: Supplementary file 1 [file cells-09-00779-s001.zip › Supplementary materials/FileS4.zip!/FileS3/TFs - Results/Summary Sheets - Differences/Reports/Sprint.2.20..vs.Zhongwan.6.25..pdf]
